# Efficacy of the fully automated oral care robot with adjunctive MA-T gel for dental biofilm removal: A prospective crossover study

**DOI:** 10.1016/j.jobcr.2026.101526

**Published:** 2026-08-25

**Authors:** Risako Sato, Maki Sotozono, Shoji Takenaka, Hiroyuki Ishii, Yuichiro Noiri

**Affiliations:** a Division of Cariology, Operative Dentistry and Endodontics, Department of Oral Health Science, Niigata University Graduate School of Medical and Dental Sciences, Niigata, Japan; b Faculty of Science and Engineering, Waseda University, Tokyo, Japan

**Keywords:** Dental biofilm, MA-T gel, Oral care robot, Dental biofilm removal, Toothbrushing, Oral hygiene

## Abstract

**Background:**

Dental biofilm (DB) is the main cause of periodontal disease and dental caries, but individuals with impaired manual dexterity may struggle to brush effectively. Although fully automatic toothbrushing devices exist, their DB removal efficacy remains limited. The clinical efficacy of the next-generation multifunctional oral care robot (OCR) has not yet been evaluated. This study evaluated the DB removal efficacy of the OCR compared with manual and sonic toothbrushes and assessed the antibacterial effect of the Matching Transformation System (MA-T) gel used with OCR.

**Materials and methods:**

In this prospective crossover study, 10 dental professionals brushed under four conditions: manual toothbrush, sonic toothbrush, OCR, and OCR with MA-T gel (OCRM). DB removal was assessed using O'Leary's Plaque Control Record (PCR), and dental biofilm reduction rates (DBRR) were compared among the four methods. For the OCR and OCRM groups, saliva samples were collected from five randomly selected participants to assess total streptococcal and *Streptococcus mutans* (*S. mutans*) counts.

**Results:**

PCR scores decreased significantly after brushing in all groups (*p* < 0.05). Pairwise comparisons demonstrated a significantly greater DBRR for the manual toothbrush than for the sonic toothbrush (*p* < 0.05), whereas no significant differences were observed among the other brushing methods. Salivary total streptococcal counts decreased more with OCRM than with OCR (*p* < 0.05). *S. mutans* reduction did not differ significantly.

**Conclusion:**

The OCR demonstrated DB removal efficacy comparable to manual and sonic toothbrushes. OCRM resulted in a greater reduction of salivary total streptococci than OCR alone.

## 1 Introduction

Periodontal disease and dental caries are the leading causes of permanent tooth loss,^1^ with dental biofilm (DB) as their primary etiological factor.^2^^,^^3^ Daily tooth brushing is essential for DB removal.^4^ Electric toothbrushes are generally more effective than manual toothbrushes in reducing DB and improving gingival health.^5^ However, even with electric toothbrushes, users must actively guide the brush along the dental arch, and adequate DB removal may be challenging for individuals with limited manual dexterity.

In recent years, Japan's rapidly aging population has driven a rise in older adults requiring nursing care, along with a marked increase in aspiration pneumonia.^6^ Oral care has been shown to reduce the incidence of pneumonia,^7^^,^^8^ highlighting the urgent need to establish effective oral hygiene methods.^9^

The next-generation multifunctional oral care robot (OCR; g.eN®, Genics Co., Ltd., Tokyo, Japan), equipped with an automatic toothbrushing system, was developed to maintain oral hygiene with minimal assistance, independent of the user's brushing skills. Previous studies have reported that fully automatic toothbrushes are less effective in DB removal than manual toothbrushes.^10^^,^^11^ The key difference between previous devices and OCR lies in the movement of the brushes along the dental arch, which may enable more thorough and effective DB removal. However, the DB removal efficacy of OCR has not yet been clinically evaluated.

Matching Transformation System (MA-T) is an antimicrobial system that generates chlorine dioxide in the presence of bacteria, with high safety due to the absence of active agents prior to use.^12^^,^^13^ Previous studies have shown that MA-T reduces bacterial viability within established biofilms, including polymicrobial DBs.^13^ We focused on MA-T mouth-clean gel® (MA-T gel; Earth Corporation, Tokyo, Japan) and investigated its combined use with OCR. The novelty of this study lies in the simultaneous application of OCR's mechanical cleaning effect and the antimicrobial action of MA-T gel to reduce oral bacterial levels.

The primary objective of this study was to evaluate and compare the DB removal efficacy of OCR with that of a manual toothbrush and a conventional electric (sonic) toothbrush. The secondary objective was to investigate whether the combined use of OCR and MA-T gel could further reduce salivary total streptococci and *S. mutans* levels compared with OCR alone.

## 2 Materials and methods

This study was designed as a prospective crossover study. The protocol was approved by the institutional ethics committee (Approval No. 2023-0329), and written informed consent was obtained from all participants. The study was conducted in accordance with the Declaration of Helsinki.

### 2.1 OCR device

**Fig. 1:**
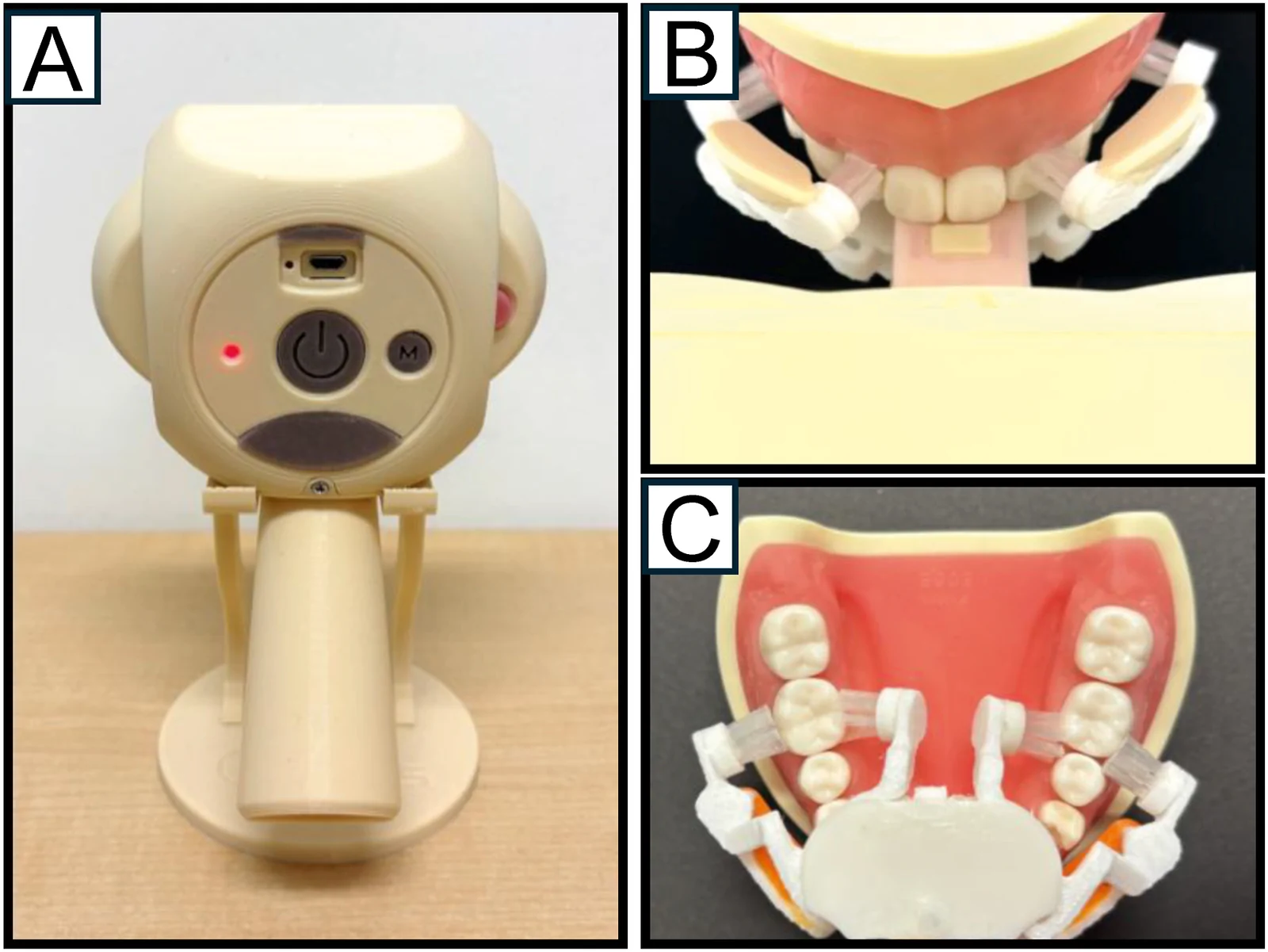
The OCR device and its intraoral application. The main unit (A) and its placement on the maxilla (B) and mandible (C).

The device (Fig. 1A) consists of detachable mouthpieces and separate brush units for the maxillary (Fig. 1B) and mandibular (Fig. 1C) arches. The adjustable mouthpiece is occluded by the anterior teeth, and the maxillary and mandibular brush units are attached and used sequentially. The base of each brush rotates 360° clockwise while moving along the dental arch, mimicking the Fones brushing technique.^14^

### 2.2 Participants

**Fig. 2:**
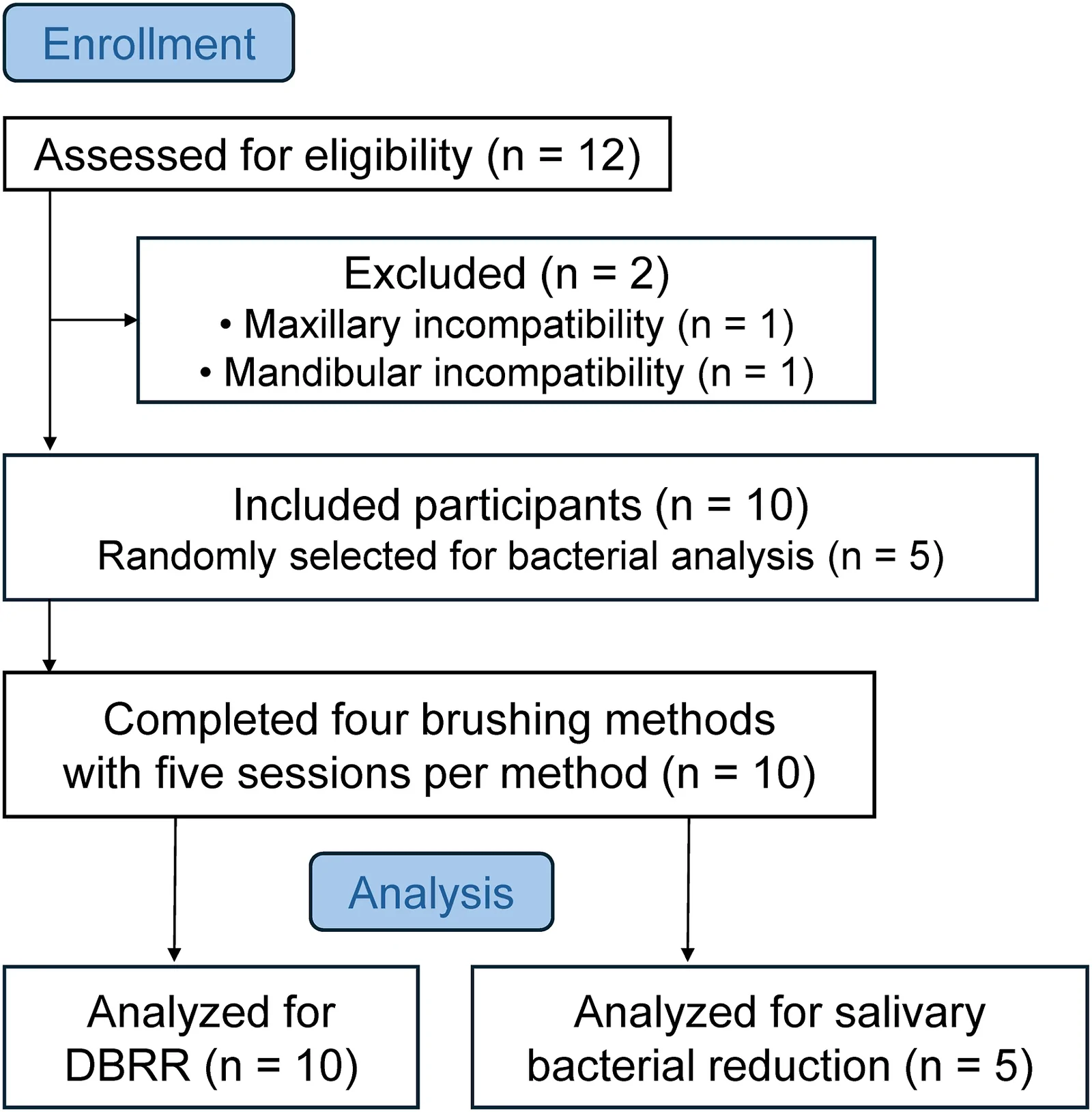
Flow diagram of participant recruitment, allocation, and analysis.

Twelve dentists and postgraduate dental students from Niigata University Medical and Dental Hospital were enrolled. Two participants were excluded because the device did not fit their dental arch (one maxillary and one mandibular incompatibility) (Fig. 2).

Inclusion criteria: age ≥18 years, ≥20 natural teeth, at least eight fully erupted teeth in each arch, and ability to bite a mouthpiece.

Exclusion criteria: partially erupted teeth, dental caries, or restorations covering >50% of the tooth surface.

### 2.3 Study protocol

This prospective crossover study compared four toothbrushing methods, with each participant completing five sessions per method (total, 200 datasets).

Occlusal characteristics (Angle's classification, overjet, and overbite), dental crowding, history of orthodontic treatment, and smoking status were recorded before the study.

Participants were instructed not to perform any oral hygiene procedures before each study visit and attend the visit within 1 h after eating. Repeated assessments within the same brushing method were performed on separate study visits at intervals of at least 3 days. Transitions between different brushing methods were generally separated by approximately 1 week. Four brushing methods—manual, sonic, OCR, and (OCR + MA-T) OCRM—were performed in a fixed order.

The manual toothbrush (BUTLER 211®; Sunstar Inc., Osaka, Japan) was used for 3 min, and the sonic toothbrush (STAGE ONE K10®; AMATERUS Co., Ltd., Tokyo, Japan) for 2 min according to the manufacturer's instructions. OCR was used according to the manufacturer's recommendations: 45 s for each of the maxillary and mandibular arches (total, 90 s). For OCRM, MA-T gel (not toothpaste) was applied to all brush components before brushing, and the same OCR protocol was followed.

DB was disclosed using 2TONE® (Kuraray Noritake Dental Inc., Tokyo, Japan), and baseline O'Leary plaque control record^15^ (PCR) scores were recorded. Participants then brushed according to the assigned protocol without toothpaste, and post-brushing PCR scores were assessed.

### 2.4 Salivary testing

Five participants were randomly selected for salivary bacterial analysis using a computer-generated randomization list created with R software (version 4.4.0; R Foundation for Statistical Computing, Vienna, Austria). Stimulated saliva samples were collected before and after brushing using the Saliva-Check Lab® system (GC Corporation, Tokyo, Japan). Participants chewed the gum provided with the kit for 5 min to stimulate saliva production. The collected saliva samples were submitted to the GC Oral Check Center (GC Corporation) for quantitative polymerase chain reaction analysis. Total streptococci and *S. mutans* levels were reported as copies/mL.

### 2.5 Outcomes

The primary outcome was DB removal rate (DBRR) calculated from PCR scores before and after brushing. Each tooth surface was assessed at four sites (mesial, distal, buccal, and palatal/lingual), and PCR was defined as the percentage of DB-positive sites. All 200 PCR datasets were included in the analysis Three dentists with at least five years of clinical experience performed the examinations and were calibrated using five representative cases before the study. The inter-examiner reliability assessed using the single-measure intraclass correlation coefficient was 0.87. Examiners were not blinded to the intervention allocation.

DBRR was calculated as follows:

$$\text{DBRR}\;\left(\%\right)=\frac{\text{Pre}\;\text{brushing}\;\text{PCR}\;\left(\%\right)-\text{Post}\;\text{brushing}\;\text{PCR}\;\left(\%\right)}{\text{Pre}\;\text{brushing}\;\text{PCR}}\times 100$$

The secondary outcome was the reduction rate of salivary total streptococci and *S. mutans* under two OCR conditions (with and without MA-T). Raw bacterial counts (copies/mL) were analyzed without logarithmic transformation. Five measurements were obtained for each participant; to reduce the influence of extreme values and measurement variability, the highest and lowest values were excluded, and the remaining three were used to calculate reduction rates.

The bacterial reduction rates were calculated as follows:

$$\text{Reduction}\;\text{Rate}\;\left(\%\right)=\frac{\text{Pre}\;\text{brushing}\;\text{bacterial}\;\text{count}-\text{Post}\;\text{brushing}\;\text{bacterial}\;\text{count}}{\text{Pre}-\text{brushing}\;\text{bacterial}\;\text{count}}\times 100$$

### 2.6 Statistical analysis

This study was designed as an exploratory proof-of-concept investigation; therefore, no a priori sample size calculation was performed. Statistical analyses were performed using IBM SPSS Statistics (version 28.0; IBM Corp., Armonk, NY, USA). After confirming normality using the Shapiro–Wilk test, pre- and post-brushing PCR values were compared within each group using paired *t*-tests. For each participant, DBRR values obtained from the five sessions for each brushing method were averaged before statistical analysis. DBRR (%) was compared among the four brushing methods using one-way repeated-measures analysis of variance (ANOVA). Sphericity was assessed using Mauchly's test, and Greenhouse–Geisser corrections were applied when the assumption of sphericity was violated. Effect sizes for repeated-measures ANOVA were reported as partial eta-squared (η^2^p). Pairwise comparisons were performed using Bonferroni-adjusted tests. Bacterial reduction rates (%) between OCR and OCRM were compared using paired *t*-tests. Statistical significance was set at *p* < 0.05 for all analyses.

## 3 Results

Ten participants completed all four brushing methods and were included in the DBRR analysis. Five participants were randomly selected for salivary bacterial analysis (Fig. 2). Baseline characteristics of the participants are summarized in Table 1.

**Table 1:** Baseline characteristics of the study participants (n = 10).

Characteristic	Value
Age, years	29.1 ± 3.62 (25–35)
Sex	Male 5 (50%); Female 5 (50%)
Number of teeth	29.6 ± 1.9 (26–32)
DMFT	4 (0-16)
Smoking status	Yes 0 (0%); No 10 (100%)
Orthodontic history	Yes 4 (40%); No 6 (60%)
Anterior crowding	Yes 1 (10%); No 9 (90%)
Angle classification (Right)	I 6 (60%); II 2 (20%); III 2 (20%)
Angle classification (Left)	I 6 (60%); II 1 (10%); III 3 (30%)
Overbite, mm	3.5 ± 1.28 (0–6)
Overjet, mm	3.21 ± 1.31 (0–6)

Abbreviations: DMFT, decayed, missing, and filled teeth; SD, standard deviation.

Supplementary Fig. S1 shows oral photographs comparing DB accumulation before and after toothbrushing.

DBRR is presented in Table 2, and baseline and post-brushing PCR values are shown in Supplementary Table S1. DBRR was highest in the anterior teeth, followed by the full mouth, and lowest in the posterior teeth. In all groups, post-brushing PCR values were significantly lower than baseline.

**Table 2:** Dental biofilm removal rates (DBRR) according to brushing method.

Brushing method	Full mouth (%)	Anterior teeth (%)	Posterior teeth (%)
Manual toothbrush	71.99 ± 27.82	79.74 ± 16.42	67.14 ± 20.17
Sonic toothbrush	52.12 ± 23.23	57.78 ± 27.41	49.80 ± 23.42
OCR	61.44 ± 17.07	65.60 ± 20.77	58.78 ± 19.27
OCR + MA-T	63.26 ± 18.02	70.53 ± 18.27	58.85 ± 19.90

DBRR were calculated from Plaque Control Record (PCR) values obtained before and after tooth brushing. Values are presented as mean ± SD.

Abbreviations: MA-T, MA-T gel; OCR, oral care robot; SD, standard deviation.

Mauchly's test indicated violation of the sphericity assumption; therefore, Greenhouse–Geisser corrections were applied. One-way repeated-measures ANOVA revealed a significant effect of brushing method on DBRR for the full mouth (*F* = 10.113, *p* = 0.002, partial η^2^ = 0.529), anterior teeth (*F* = 7.668, *p* = 0.004, partial η^2^ = 0.460), and posterior teeth (*F* = 7.392, *p* = 0.009, partial η^2^ = 0.451).

Pairwise comparisons are summarized in Table 3. Bonferroni-adjusted pairwise comparisons revealed that the manual toothbrush (71.99 ± 27.82%) achieved significantly higher DBRR than the sonic toothbrush (52.12 ± 23.23%) in the full mouth, anterior, and posterior teeth. No significant differences were observed between the OCR (61.44 ± 17.07%), OCRM (63.26 ± 18.02%), and sonic toothbrush groups, or between the OCR and OCRM groups.

**Table 3:** Pairwise comparisons of dental biofilm removal rates (DBRR) among brushing methods.

Methods 1	Methods 2	Difference in reduction	95% CI
Full mouth			

Manual toothbrush	Sonic toothbrush	19.87*	9.00, 30.74
	OCR	10.55	−3.45, 24.55
	OCR + MA-T	8.73	−4.57, 22.03
Sonic toothbrush	OCR	−9.32	−23.59, 4.95
	OCR + MA-T	−11.14	−24.41, 2.13
OCR	OCR + MA-T	−1.82	−6.34, 2.69

Anterior teeth			

Manual toothbrush	Sonic toothbrush	22.08*	3.67, 40.49
	OCR	14.24	−2.13, 30.62
	OCR + MA-T	9.31	−1.86, 20.48
Sonic toothbrush	OCR	−7.83	−28.43, 12.77
	OCR + MA-T	−12.77	−29.84, 4.30
OCR	OCR + MA-T	−4.94	−13.07, 3.20

Posterior teeth			

Manual toothbrush	Sonic toothbrush	18.33*	8.02, 28.64
	OCR	8.16	−8.62, 24.95
	OCR + MA-T	8.21	−8.82, 25.23
Sonic toothbrush	OCR	−10.17	−23.30, 2.97
	OCR + MA-T	−10.12	−22.19, 1.94
OCR	OCR + MA-T	−0.04	−6.21, 6.29

Differences in DBRR are presented as mean differences with 95% confidence intervals (CI). Pairwise group comparisons were performed using one-way repeated-measures analysis of variance (ANOVA) followed by Bonferroni-adjusted pairwise comparisons. * indicates a statistically significant difference (*p* < 0.05).

Abbreviations: MA-T, MA-T gel; OCR, oral care robot.

**Fig. 3:**
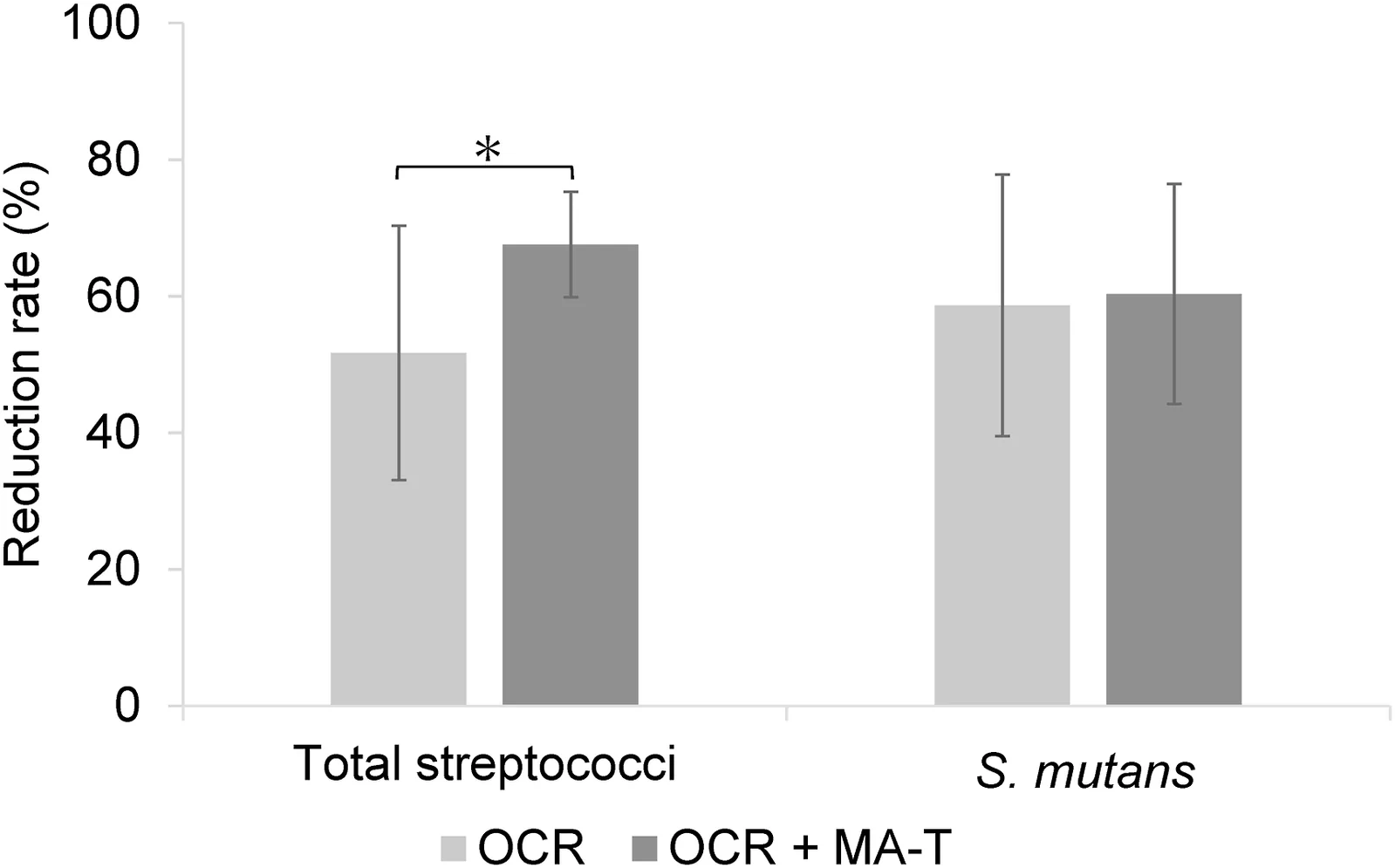
Reduction rates of total streptococci and *S. mutans* following use of OCR with and without MA-T gel. Data are presented as mean ± standard deviation. Pairwise comparisons were performed using paired *t*-tests. * indicates a statistically significant difference (*p* < 0.05). Abbreviations: MA-T, MA-T gel; OCR, oral care robot.

The reduction rate of total streptococci was significantly higher in the OCRM (67.59 ± 7.73%) than in the OCR (51.69 ± 18.63%), whereas no significant difference was observed in *S. mutans* reduction between the two groups (Fig. 3).

No adverse events were observed during the study.

## 4 Discussion

Previous reviews reported mean DBRR values of 42% for manual toothbrushes^16^ and 46% for electric toothbrushes.^5^ In the present study, all brushing methods achieved higher DBRR values than those previously reported. Although the manual toothbrush showed better results than the electric toothbrush, which contrasts with previous reports,^5^ this may reflect that the participants were dental professionals who routinely used manual toothbrushes and possessed a high level of toothbrushing skill.^17^^,^^18^ In addition, the brushing duration was longer for the manual toothbrush (3 min) than for the sonic toothbrush (2 min), which may also have influenced the observed difference. Furthermore, participants were aware that their oral hygiene performance was being evaluated, which may have contributed to the generally higher DBRR values observed in this study compared with previous reports. OCR and OCRM did not differ significantly from either the manual or sonic toothbrushes and achieved DBRR values of approximately 61% and 63%, respectively.

Previous studies have indicated that insufficient coverage of the posterior teeth and gingival margin is a limitation of fully automatic toothbrushes,^19^ which may restrict their DB removal efficacy. In this study, the OCR and OCRM groups demonstrated stable DBRR of approximately 59% in the posterior region. This value exceeded the mean DBRR values previously reported for manual toothbrushes (42%)^16^ and electric toothbrushes (46%),^5^ suggesting that OCR can achieve effective DB removal even in posterior areas. In addition, OCR completed the brushing procedure in 90 s. Considering that posterior cleaning is often time-consuming, the short brushing time required by OCR may help reduce the burden associated with oral care.^20^

The OCRM group demonstrated a significantly greater reduction in total streptococci than OCR alone, which is consistent with previous studies reporting the antimicrobial activity of MA-T against polymicrobial DBs.^13^ These findings suggest that the addition of MA-T gel may enhance the reduction of salivary total streptococci.

In contrast, no significant difference was observed in *S. mutans* reduction between the two groups. The low baseline level of *S. mutans* may explain the lack of a significant reduction. Future studies should investigate the effect of OCR combined with MA-T gel in individuals with higher baseline *S. mutans* levels.

A major strength of this study is that OCR, a fully automatic toothbrush, demonstrated DB removal efficacy comparable to that of manual and sonic toothbrushes. Furthermore, the repeated-measures design allowed DB removal efficacy to be evaluated across multiple brushing sessions, providing a more stable assessment than a single brushing episode. The study also assessed the adjunctive use of MA-T gel and its effect on salivary bacterial reduction, providing preliminary evidence for combining mechanical cleaning and antimicrobial approaches.

One limitation of this study is the DB quantitative assessment method. Because the OCR lacks an occlusal surface brush, PCR—which assesses DB at the gingival margin—was selected. Various DB measurement techniques exist and may yield different results.^16^ Incorporating an occlusal surface brush into the OCR remains a challenge for future development.

Another limitation is device compatibility. Two participants who met the manufacturer's criteria were unable to use the device: one in the maxillary arch and one in the mandibular arch. Both had average-sized arches, suggesting that the arch form and occlusion may influence device compatibility.

The fixed intervention order may have introduced carryover and learning effects across study visits. Furthermore, the lack of examiner blinding may have introduced detection bias during PCR assessment. These factors may have influenced the observed treatment effects. In addition, this study was conducted as a small proof-of-concept study intended to provide an initial assessment of OCR performance. The participants were limited to dental professionals with good oral hygiene and high toothbrushing skills. Therefore, the findings should be interpreted with caution, and their generalizability to the broader population may be limited. Moreover, the study evaluated only immediate DB removal and salivary bacterial reduction and did not assess long-term outcomes, gingival health, patient comfort, or usability. Further studies involving larger and more diverse populations are warranted to confirm the clinical applicability of OCR.

## 5 Conclusion

OCR demonstrated DB removal efficacy comparable to that of manual and sonic toothbrushes. In addition, the adjunctive use of MA-T gel resulted in a greater reduction of salivary total streptococci than OCR alone. Further studies are warranted to confirm these findings in larger and more diverse populations.

## Patient/guardian consent

Written informed consent was obtained from all participants for participation in this crossover interventional study and for the publication of anonymized images.

## Ethical clearance

Ethical approval for this study was obtained from the institutional ethics committee of Niigata University (Approval No. 2023-0329; dated April 9, 2024).

## Data statement

All data generated or analyzed during this study are included in this published article and its supplementary information files.

## Human statement

The study protocol was approved by the Ethics Committee of Niigata University (Approval No. 2023-0329). All procedures were performed in accordance with the Declaration of Helsinki and relevant ethical guidelines. Written informed consent was obtained from all participants.

## Sources of funding

This study was supported by a donation and MA-T gel provided by Earth Corporation. The sponsor had no role in the study design; data collection, analysis, or interpretation; manuscript preparation; or the decision to submit the manuscript for publication.

## Declaration of competing interest

The authors declare the following financial interests/personal relationships which may be considered as potential competing interests: Yuichiro Noiri reports financial support and equipment, drugs, or supplies were provided by Earth Corporation. If there are other authors, they declare that they have no known competing financial interests or personal relationships that could have appeared to influence the work reported in this paper.

## Figures and Tables

**Figure:**
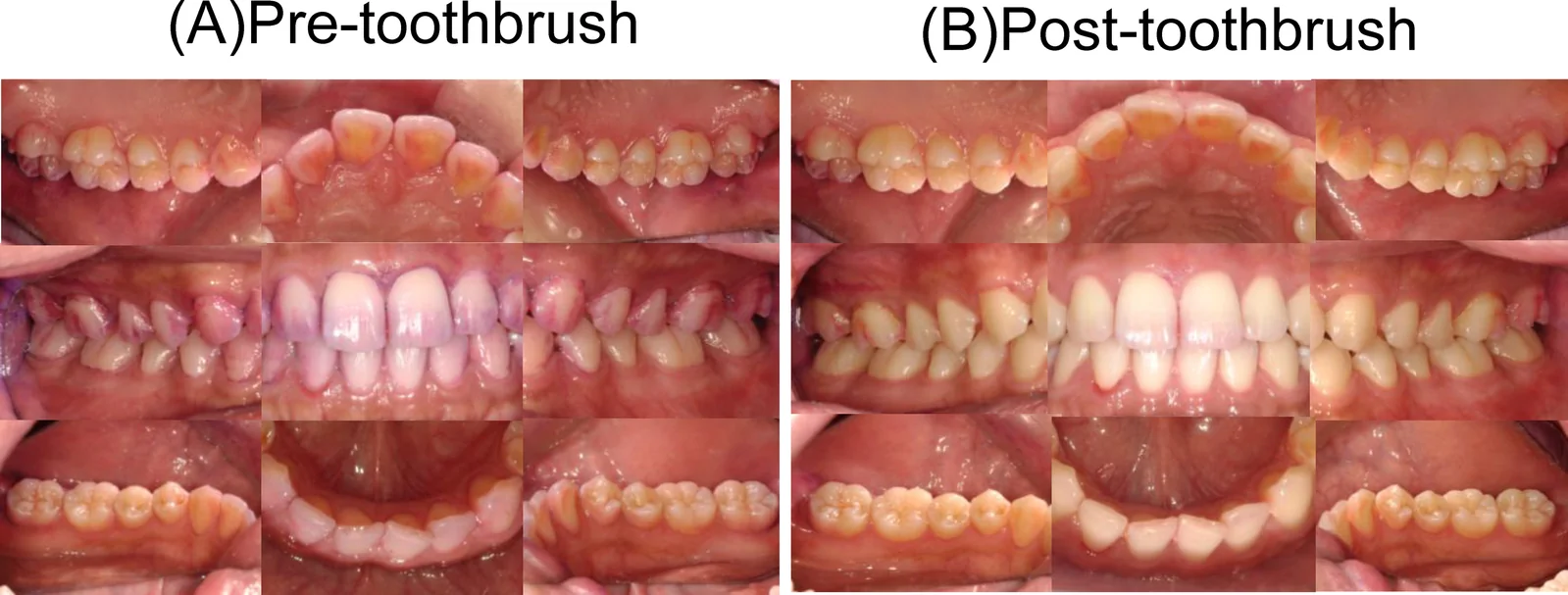
Supplementary Figure S1. Oral photographs comparing plaque accumulation before (A) and after (B) toothbrushing using OCR with a disclosing agent.
